# 561 Ethnicity Influences Outcomes of Adult Burn Patients

**DOI:** 10.1093/jbcr/irac012.189

**Published:** 2022-03-23

**Authors:** Nicola DiPaolo, Ian F Hulsebos, Jeremy Yu, Justin Gillenwater, Haig A Yenikomshian

**Affiliations:** Keck School of Medicine of USC, Los Angeles, California; Keck School of Medicine of USC, Pasadena, California; University of Southern California, Los Angeles, California; USC/LAC+USC Medical Center, Los Angeles, California; USC/LAC+USC Medical Center, Los Angeles, California

## Abstract

**Introduction:**

Outcomes of burn survivors is a well-studied field of research for burn providers; however, there has been little data comparing the outcomes of burn survivors by ethnicity. This study seeks to identify any disparities in burn outcomes of broad ethnic groups. Adjustment was made for demographic, social and pre-hospital clinical factors to help isolate ethnic disparities that might not be explainable by other factors.

**Methods:**

A retrospective chart review of an American Burn Association verified burn center identified adult inpatient admissions from 2015 to 2019 with documented insurance status. A total of 1142 patients were categorized by recorded primary ethnicity: 142 Black (or African American), 72 Asian, 479 Hispanic (or Latinx), 90 white, 215 other, and 144 patients whose race or ethnicity was not indicated. Firth logistic regression was used to study the relationship between ethnicity and each of several binary outcomes. Zero-truncated negative binomial regression was used to examine hospital length of stay (LOS) and intensive care unit LOS. Adjustment was made for several confounders (age, gender, homelessness, primary insurance type, diabetes, inhalation injury, primary burn depth, percentage of total body surface area injured) to clarify the statistical effect of ethnicity. The specific adjustment set used depended on the outcome type or frequency.

**Results:**

Relative to white patients, surviving Black patients had an estimated 29% higher average hospital LOS (ratio 1.29; 95% CI 1.01-1.64; unadjusted P=.04). Had the average surviving patient in this sample been Black, their hospital stay would be 2.7 days longer (95% CI 0.1-5.4). Relative to white patients, the odds of being discharged home with or without services, or to hospice care, were an estimated 123% higher for Hispanic patients (OR 2.23; 95% CI 1.28-3.88; unadjusted P=.005). Compared with non-Hispanic ethnicity, Hispanic ethnicity was associated with a 44% decrease in the odds of discharge to acute care, inpatient rehabilitation, or a ward outside the burn unit (OR 0.56; 95% CI 0.34-0.92; unadjusted P=.022). Black and Hispanic patients had a higher relative chance of having publicly assisted insurance, versus private insurance, than their white counterparts (P=.041, P=0.011 respectively).

**Conclusions:**

Even when controlling for burn severity, age, and other factors, Black patients had longer hospital stays. Hispanic patients were more likely to be discharged to home or to hospice care. The causes of these disparities are indeterminate. They may stem from socioeconomic status not entirely accounted for, ethnic differences in comorbidity related to stressors, or inequity in health care delivery or insurance coverage.

